# Impact of Inflammatory Markers and Senescence-Associated Secretory Phenotype in the Gingival Crevicular Fluid on the Outcomes of Periodontal Regeneration

**DOI:** 10.3390/ijms25126687

**Published:** 2024-06-18

**Authors:** Giacomo Baima, Federica Romano, Francesco Franco, Ilaria Roato, Federico Mussano, Giovanni Nicolao Berta, Mario Aimetti

**Affiliations:** 1 Department of Surgical Sciences, C.I.R. Dental School, University of Turin, 10026 Turin, Italy; federica.romano@unito.it (F.R.); ilaria.roato@unito.it (I.R.); federico.mussano@unito.it (F.M.); mario.aimetti@unito.it (M.A.); 2 Department of Clinical and Biological Sciences, University of Turin, 10026 Turin, Italy; francesco.franco@unito.it (F.F.); giovanni.berta@unito.it (G.N.B.)

**Keywords:** cytokines, gingival crevicular fluid, inflammation, periodontal regeneration, senescence

## Abstract

The aim of this study was to test the molecular expression profile (senescence-associated secretory phenotype; SASP) in gingival crevicular fluid (GCF) prior to surgery in relation to the distribution of clinical success of periodontal regeneration. Forty consecutive patients presenting sites with residual probing pocket depth (PPD) ≥ 6 mm and intrabony defects ≥ 3 mm were treated through a minimally invasive surgical technique. Pre-operatively, GCF was sampled for inflammatory biomarker analysis related to SASP [interleukin (IL)-1β, IL-6, and IL-12; matrix-metalloproteinases (MMP)-8 and -9]. Better or worse responders were classified depending on the achievement of a composite outcome measure at 1-year [COM; PPD ≤ 4 mm and clinical attachment gain (CAL) gain ≥ 3 mm]. Correlation analyses and logistic regression models were performed. Periodontal regeneration led to significant improvements in mean clinical and radiographic parameters. Teeth achieving COM presented significantly lower amounts of SASP factors compared with non-successful teeth. Higher CAL gain, PPD reduction, and radiographic bone fill were negatively correlated with IL-1β and MMP-8 and -9 (*p* < 0.001), while IL-12 showed a direct relationship with CAL gain (*p* = 0.005) and PPD reduction (*p* = 0.038). Sites expressing higher SASP expression in the GCF before periodontal regeneration achieved worse clinical and radiographic outcomes.

## 1. Introduction

The multifactorial imbalance in the relationship between host and parasites seen in advanced periodontal lesions results in heterogeneous patterns of periodontal attachment and alveolar bone destruction [1,2]. One specific type of bone defect anatomy found in severe periodontitis stages is the infrabony defect, where the pocket base lies apical to the alveolar crest [3]. To address these anatomical deformities, recent guidelines approved by the European Federation of Periodontology (EFP) recommend periodontal regeneration [4,5].

Periodontal regeneration techniques employ various approaches to stimulate the body’s natural healing mechanisms, and reform damaged periodontal tissues, primarily relying on three biological principles: adequate scaffolds, molecular mediators, and cells [6,7,8]. Prognostic factors related to the patient, defect, and site significantly impact regenerative therapy outcomes [9,10]. These may include plaque/inflammatory control, systemic factors, local anatomy, surgical techniques, and inherent wound healing potential [11,12]. Despite the fact that no current standard methods exist, the latter aspect could be investigated by detecting the level of expression of certain pro-inflammatory or pro-resolving molecules in the gingival crevicular fluid (GCF) [13,14].

Recently, senescent cells within periodontal tissues have been described in animal and human models, potentially impairing the stem cell reservoir [15,16]. Senescent cells not only have limited intrinsic potential for tissue healing but also contribute to negative effects on the surrounding niche and chronic systemic inflammation through the secretion of the senescent-associated secretory phenotype (SASP) [17,18,19]. SASP comprises proinflammatory cytokines, chemokines, and proteases, such as interleukin (IL)-1β, IL-6, IL-12, tumor necrosis factor-α, and matrix metalloproteases (MMPs), which alter the surrounding microenvironment and impact on the healing outcomes [20,21].

Based on these premises, it was hypothesized that higher site-specific clinical inflammation and increased SASP secretion in the GCF might negatively affect periodontal regenerative procedures. Therefore, the aim of this study was to assess the impact of inflammation-related changes in the periodontal microenvironment and SASP expression in the GCF on the clinical and radiographic outcomes of periodontal regeneration.

## 2. Results

### 2.1. Patient Characteristics and Clinical Outcomes

Forty patients (24 females/16 males; mean age: 57.5 ± 9.4 years) providing one experimental site each were consecutively enrolled. Their characteristics, both at patient- and tooth-level, are summarized in Table 1. The distributions of intrabony defects according to teeth were 32.5% anterior, 40.0% premolar, and 27.5% molar. No major adverse outcomes (acute infections; tooth loss) were observed in any of the patients after the treatment. As summarized in Table 2, periodontal regeneration yielded significant changes at 1 year in terms of BoP and PPD reduction, as well as CAL gain. Overall, 25 patients (62.5%) obtained COM for periodontal regeneration, whereas 15 patients (37.5%) did not.

### 2.2. Association between Inflammatory Markers and Clinical Parameters at Baseline

Before surgery (T0), GCF levels of IL-1β and MMP-9 were both negatively related to PPD (*p* < 0.001 and *p* = 0.032, respectively) and CAL (*p* = 0.002 and 0.036, respectively); MMP-8 negatively correlated with PPD (*p* = 0.004); whereas IL-6 and IL-12 did not correlate with any clinical parameters. Regarding BoP, only MMP-8 was statistically significantly higher in sites presenting clinical inflammation (*p* = 0.02). Age and gender did not influence the cytokine expression profile.

### 2.3. Impact SASP on Periodontal Regeneration

In relation to the influence of an increased inflammatory expression profile on the outcomes of periodontal regeneration (Table 3), a statistically negative correlation was noted between the levels of IL-1β, MMP-8, and -9 with CAL gain, PPD reduction, and radiographic bone fill observed at the 12-month post-surgery (all *p* < 0.001). The opposite trend was observed for IL-12, which positively correlated with CAL gain (*p* = 0.005) and PPD reduction (*p* = 0.048). No significant associations were observed for IL-6.

**Figure 1:**
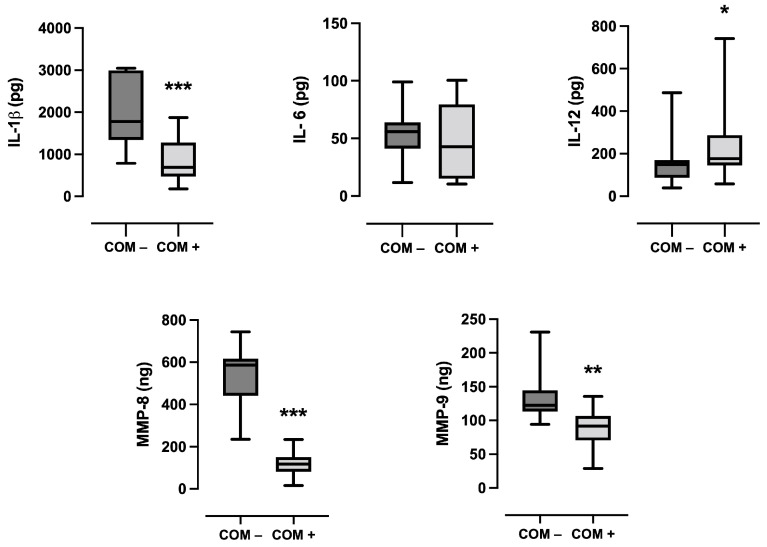
Box plots showing the total amount of inflammatory mediators between patients who achieved or did not achieve the clinical composite outcome of periodontal regeneration (COM). The box represents the median, 25%, and 75% percentiles; the whiskers represent data within 10% and 90% percentiles. * *p* < 0.05; ** *p* < 0.01; *** *p* < 0.001.

Table 4 summarizes clinical and biomolecular data stratified for sites achieving or not achieving COM, respectively. Among the clinical variables, a higher baseline PPD was significantly associated with COM achievement (*p* = 0.007), whereas all the other factors did not. Among sites achieving COM, levels of IL-1β (*p* < 0.001), MMP-8 (*p* = 0.001), and MMP-9 (*p* < 0.001) were significantly lower compared with sites that did not reach successful regenerative outcomes, whereas IL-12 was significantly higher (*p* = 0.015) (Figure 1).

Finally, logistic multivariate regression models were built with COM as the dependent variable. Despite reducing the odds for successful regeneration, clinical inflammation (BoP) at baseline did not reach statistical significance (*p* = 0.169). Interestingly, higher IL-1β and MMP-8 baseline levels were significantly associated with a lower probability of achieving COM (both *p* < 0.05). Also, higher MMP-9, together with lower IL-12 values, were associated with lower odds of achieving successful outcomes of periodontal regeneration (*p* = 0.007 and *p* = 0.038, respectively) (Table 5).

## 3. Discussion

The aim of the present pilot study was to evaluate how pre-surgical patterns of molecular inflammation in the GCF can impact the outcomes of periodontal regeneration. The results showed a negative influence of the expression of the majority of SASP factors analyzed on 1-year CAL gain, PPD reduction, and radiographic bone fill. A combined logistic regression model implementing IL-1β together with MMP-8, as well as MMP-9 together with IL-12, showed the best predictive ability for final regenerative clinical outcomes. Prior studies suggest that minimizing both the bacterial biofilm load and the site-specific inflammatory burden is crucial before undergoing regenerative periodontal treatment [22,23,24]. Indeed, higher plaque indexes and lack of inflammation control prior to surgical therapy have been associated with worse clinical outcomes in previous investigations [11,25]. A previous study from our group showed how site-specific clinically and molecularly assessed inflammation had a negative impact on early wound healing after the performance of minimally invasive flaps [23]. However, there is limited evidence of their long-term healing outcomes. In the present analysis, we did not find a statistically significant association between clinically assessed inflammation and final COM. This aspect can be ascribed to the fact that the study might not have been powered enough to detect differences in BoP or that only deep bleeding was assessed [26]. Indeed, superficial bleeding could be more related to early wound healing after surgery, and this peculiar difference might be considered in future investigations.

The novelty of the present study consists in considering an array of relevant inflammation-related molecules as proxies for the inflammaging/senescent state of the subgingival environment prior to periodontal regeneration. The concept of senescence has been recently implicated in periodontitis pathogenesis through chronic exposure to pathogen-associated molecular patterns or reactive oxygen species [18,19]. Senescing cells exhibit SASP, i.e., a multitude of secretory proteins, including inflammatory and matrix-modeling signaling molecules, released with autocrine and paracrine effects [27]. In a pro-senescent microenvironment, it is plausible that the inner regenerative potential of the periodontium is impaired due to the depletion of the stem cell reservoir and the altered paracrine secretion of growth factors/pro-inflammatory molecules [16,28,29]. Indeed, a higher SASP expression in the GCF from periodontal intrabony defects compared with healthy control sites has been described recently [30]. Based on this background, it was hypothesized that SASP expression levels could affect the long-term results of periodontal regeneration. From a more comprehensive perspective, SASP includes pro-inflammatory cytokines such as IL-1α/β, IL-6, and IL-8; chemokines; proteases encompassing MMPs and activators of plasminogen; growth factors such as VEGF, TGF-β, and GM-CSF; and extracellular vesicles [31]. Among these markers, we have employed the array of molecules that had a stronger scientific validation as diagnostic and prognostic markers of periodontitis [32,33]. Notably, statistically significant positive associations were established between pre-surgical levels of IL-1β, MMP-8, and -9 in the GCF and clinical and radiographic parameters at the 12-month follow-up. These observations are in agreement with what is biologically reasonable to expect since these molecules are widely recognized as inflammatory mediators, acting as key active players in the etiopathogenetic process of periodontitis [34]. Specifically, IL-1β and IL-6 are the principal pro-inflammatory cytokines which contribute to the immune T-helper 17 mediated dysregulated response underlying periodontitis pathogenesis [35]. Regarding IL-1β, the literature is quite consistent in indicating its decrease after successful non-surgical therapy while remaining high in sites showing clinical progression [36,37]. Conversely, data regarding IL-6 as an accurate biomarker are more controversial. Indeed, the levels of this cytokine may reflect more the confounding effect of other patient-level determinants, such as age, gender, and body mass index [38]. Indeed, no significant correlations were found between this cytokine and any of the investigated outcomes. Mainly released by neutrophil and macrophage populations [39], MMPs are regarded as the effectors of the unbalanced matrix degradation during the active phases of periodontitis, being useful diagnostic and prognostic markers for periodontitis [40]. These zinc-dependent proteases were also detected at higher levels in aged periodontal tissues, being a potential proxy for the local inflammaging status of tissues [41]. Finally, IL-12 was found to be positively correlated with PPD reduction in our study. In previous works, the amount of this molecule was found to be similar to that in healthy controls, as well as to decrease after therapy [42,43]. Despite the pathophysiologic role of this cytokine is still controversial, IL-12 is strongly associated with a T helper 1 response, mediating the clearance of pathobionts, suppressing osteoclastic activity, and potentially exhibiting both a proinflammatory and immunoregulatory role in periodontal pathophysiology [35,44]. Interestingly, a combination of IL-1β together with MMP-8, and of IL-12 together with MMP-9 increased the probability of identifying more successful cases. If corroborated, these results could find a clinically relevant applicability if incorporated in a chair-side testing instrument to predict the potential of healing of a determined site.

Comparison of these present data with the existing literature is challenging. Indeed, most of the authors have analyzed the effect of periodontal therapy on the initial variation of interleukins and MMPs, confirming a significant decrease for IL-1β, IL-6, and MMP-8/9 [23,45]. To the best of our knowledge, this is the first study in which the GCF markers were hypothesized as independent variables at the baseline, influencing the long-term clinical results of periodontal regeneration therapy. Another strength is related to the use of multiplex immunoassay technology for the simultaneous assessment of different biomarkers using a very small amount of GCF samples [33,36,46,47]. Some limitations also need to be acknowledged, mainly related to the absence of previous data to calculate the sample size, which might have limited the power of other predictors of periodontal regeneration [12,48]. Although it partially affects external validity, we have used strict eligibility criteria to include comparable cases in terms of clinical and surgical characteristics to control for confounding factors. The generalizability of these findings could be further tested by enrolling patients with risk factors linked to less favorable healing outcomes, including individuals who smoke and those with diabetes. Also, it would have been valuable to assess whether the ‘non-respondent’ sites still displayed at 1-year the same cytokine expression pattern, but this topic could be the subject of future investigation with GCF sampled at the revaluation.

The clinical assessment of the real senescent state of a tissue/organ is far from being comprehensively reflected by an array of expressed cytokines. However, despite the entire medical literature acknowledging the significance of cellular senescence in the pathogenesis of chronic inflammatory diseases [49,50], accurate chair-side biomarkers are currently lacking. Overall, the clinical applications of these findings in the context of periodontal regeneration and other regenerative medicine approaches remain largely unexplored. Regulating the inflammatory response and managing the release of cytokines, especially by controlling their activation or inhibition in a precise time and location, could present an important avenue for periodontal tissue engineering [51]. In this regard, further research should aim to bridge the gap between the laboratory and clinical settings. As the global population ages, the prevalence of periodontal diseases and the need for effective treatment options are increasing. This study can contribute to the advancement of periodontal regeneration techniques by shedding light on the role of the local inflammaging/senescence status. Indeed, among the other well-known factors affecting the outcomes of periodontal regeneration, there might be a significant role played by an inner biological reservoir of the patient at the site level, which we hypothesize is influenced by its background senescent state. Obtaining a closer understanding of these factors is important for developing more effective and tailored regenerative strategies based on the patient’s regenerative potential.

## 4. Materials and Methods

The protocol was approved by the Institutional Ethical Committee (protocol number 00309/2021), and it was registered on clinicaltrials.gov (NCT06354972). This study complies with the principles of the Declaration of Helsinki, and it is reported in the STROBE statement [52]. All patients provided signed informed consent before enrollment.

### 4.1. Study Design and Population

**Figure 2:**
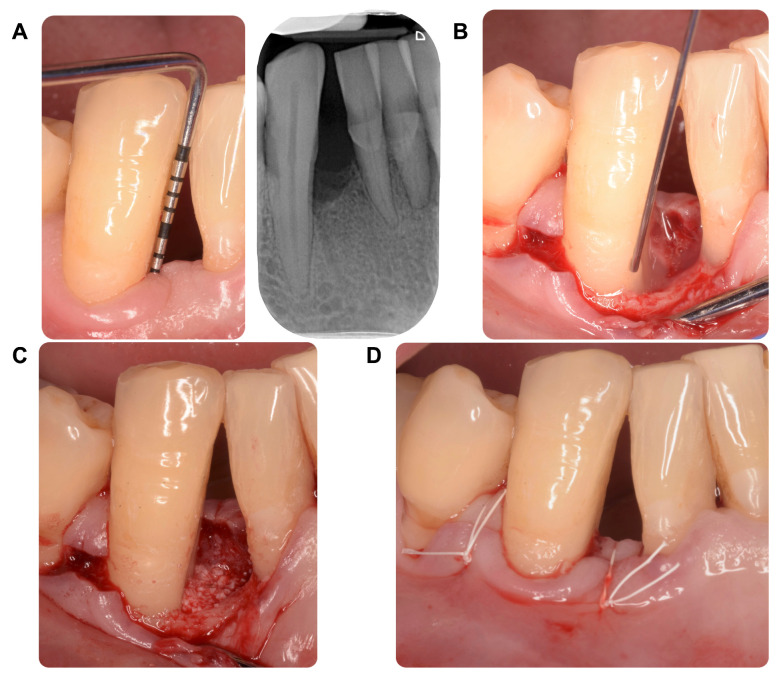
Description of the clinical procedure. Clinical and radiographic images showing the presence of a deep probing pocket depth associated with the radiographic aspect of an intrabony defect (**A**). After flap elevation, the defect is treated with a combination of enamel matrix derivatives (**B**) and bone xenograft (**C**). Eventually, the minimally invasive flap was sutured to obtain primary intention healing (**D**).

The research was designed as a prospective observational study, where consecutive patients who had undergone steps I-II of periodontal therapy were considered for inclusion between January and November 2022. One expert examiner evaluated the eligibility based on the following specific criteria. Inclusion criteria comprised: (i) diagnosis of stage III or IV periodontitis [53]; (ii) full-mouth plaque score (FMPS) and full-mouth bleeding score (FMBS) of <15%; (iii) completion of steps I–II of periodontal treatment at least 2 months priorly; (iv) tooth with residual probing pocket depth (PPD) ≥ 6 mm, BoP+; a radiographic intrabony component ≥ 3 mm, lacking furcation involvement, considered suitable for a minimally invasive procedure (MIST; [54]) (Figure 2A). Exclusion criteria encompassed: (i) age < 18 years; (ii) current smokers [55]; (iii) contraindications to surgery; (iv) systemic diseases that could impact periodontal healing (i.e., uncontrolled diabetes mellitus, severe immunodeficiencies); (v) pregnancy and lactation; (vi) a history of periodontal surgery at the experimental teeth.

### 4.2. Intervention

All experimental sites underwent a MIST procedure using surgical loupes with 3.5 to 4.7 magnification. The full-thickness flap was minimally raised both on the buccal and oral side, avoiding vertical releasing incisions. Granulation tissue was scraped from the bony surfaces of the defect, and the root was debrided using minicurettes/ultrasonic devices and chemically treated by EDTA (PrefGel, Institut Straumann AG, Basel, Switzerland). The regenerative procedure was carried out using a combination of enamel matrix derivatives (Emdogain, Institut Straumann AG) and bone xenograft (Bio-Oss, Geistlich Pharma AG, Wolhusen, Switzerland) (Figure 2B,C). The flaps were then repositioned and passively sutured (6.0; Gore-tex, WL Gore & Associated, Flagstaff, AZ, USA) (Figure 2D). After therapy, patients were prescribed analgesic medication (ibuprofen 600 mg, every 8 h for 3 days), as well as 0.20% chlorhexidine digluconate mouth rinse for 1 min (2 times/day for 2 weeks). Clinical control and wound medication were performed one week later. Sutures were removed 2 weeks after surgery. During the postoperative period, patients were advised to avoid toothbrushing and flossing in the operated area.

### 4.3. Clinical Measurements

Clinical measurements were taken at the deepest point of the selected defects by using a manual 1-mm graduated periodontal probe (PCP-UNC 15, Hu-Friedy, Chicago, IL, USA) on the day of surgery (T0) and 1 year after periodontal regenerative surgery (T1) by the same blinded examiner. To perform the intra-examiner calibration, 8 non-study patients complying with inclusion criteria were evaluated on two diverse occasions within 2 days. The percentage of agreement within 1 mm between repeated measurements of CAL and PPD was ≥96% and ≥93%, respectively. The following clinical parameters were assessed at T0 and T1: presence/absence of bacterial plaque, presence/absence of BoP, PPD, gingival recession (REC), CAL, and the width of keratinized tissue (KT). The number of prevalent bony walls of the defect was registered intrasurgically.

### 4.4. GCF Sampling

The GCF was collected from the experimental sites before any clinical examination was conducted to ensure the prevention of blood contamination. The process involved isolating the sites with cotton rolls and meticulously removing any supragingival plaque. After gently drying the areas using an air syringe, GCF samples were obtained using paper strips (PerioPaper Strips, Oraflow Inc., Plainview, NY, USA). These strips were carefully inserted into the pockets until they encountered slight resistance and were left in place for 30 s. Any strip contaminated with blood was excluded from the study. The volume of collected GCF was measured, and each GCF-containing strip was placed into separate coded sealed Eppendorf microcentrifuge tubes, each containing 100 μL of sterile phosphate-buffered saline (PBS). These samples were stored at −80 °C until further processing.

### 4.5. Multiplex Bead Immunoassay

Levels of the markers IL-1β, IL-6, IL-12, MMP-8, and MMP-9 were assessed using the highly sensitive Bio-Plex 200 Suspension Array System (Bio-Rad Laboratories S.r.l., Segrate, Milan, Italy), according to the manufacturer’s provided protocols. The sensitivity of the assay varies depending on the analytes and the kit used for their measurement. In brief, specialized anti-cytokine antibody-conjugated beads were loaded into individual wells of a 96-well plate. Following a washing step, standards and undiluted GCF samples were added to their respective wells and allowed to incubate for 30 min. Subsequently, the plates were washed, and biotin-conjugated detection antibodies were introduced. After an additional 30-min incubation and subsequent washing, streptavidin-conjugated PE was added for a 10-min interval. The resulting complexes were then solubilized by introducing Bio-Plex assay buffer to each well and subjected to analysis by the Bio-Plex Suspension Array System to determine the total quantities of each marker.

### 4.6. Statistical Analysis

The normality of these data’s distribution was assessed using the Shapiro–Wilk test. Differences in continuous variables were evaluated through paired *t*-tests or Wilcoxon tests, depending on the distribution of these data. Categorical variables were compared using the chi-square test or Fisher’s exact test as appropriate. Spearman’s and Pearson’s correlation analyses were performed to investigate associations between GCF cytokine values and clinical/radiographic outcomes, respectively. A stepwise backward logistic regression model was constructed to explore the impact of GCF biomarker levels on successful regeneration [composite outcome measure (COM): PPD ≤ 4 mm and clinical attachment level (CAL) gain ≥ 3 mm; [56]], and the results were presented as odds ratios (OR) with 95% confidence intervals (CI). For biomarkers, IL-1, IL-6, IL-12, MMP-8, and MMP-9 were grouped as low or high according to their median values [57]. Final multivariate models were corrected for the depth of the radiographic bone defect, which was the only variable significantly associated with COM in the univariate models. Statistical significance was defined as *p*-values less than 0.05. All statistical analyses were carried out using commercially available software (IBM SPSS Statistics, version 28.0.1.0).

## 5. Conclusions

A higher pre-surgical expression of IL-1β, MMP-8, and MMP-9 in the GCF negatively correlated with 1-year clinical and radiographic outcomes of periodontal regeneration, whereas IL-12 showed the opposite significant trend. These novel human findings suggest that higher SASP expression at the local levels may predict less favorable healing outcomes and emphasize the need to (i) consider these factors as biological predictors of periodontal regenerative procedures; (ii) explore novel therapeutic approaches to modulate inflammation/senescence within the periodontal microenvironment prior to surgery in order to improve its effectiveness.

## Figures and Tables

**Table 1:** Characteristics of the experimental sample at the baseline.

Variables	Values
Patient level	
Age (years; mean ± SD)	57.5 ± 9.1
Females/Males (n)	24/16
FMPS (%; mean ± SD)	10.7 ± 1.8
FMBS (%; mean ± SD)	8.1 ± 1.7
Tooth level	
Tooth type (anterior/premolar/molar; %)	32.5/40.0/27.5
Dental arch (maxilla/mandible; %)	62.5/37.5
Biotype (thin/thick; %)	17.5/82.5
Number of predominant bony walls (1/2/3; %)	0.0/50.0/50.0
KT width at buccal site (mean ± SD)	4.1 ± 1.2

FMPS, full-mouth plaque score; FMBS, full-mouth bleeding score; KT, keratinized tissue width; SD, standard deviation.

**Table 2:** Characteristics of treated sites at baseline and at 1 year after periodontal regeneration.

Variables	Baseline	1-Year	*p*-Value
Presence of plaque (%)	20.0	12.7	<0.05
BoP (%)	52.5	30.0	<0.05
PPD (mm; mean ± SD)	7.6 ± 2.2	4.1 ± 1.4	<0.01
REC (mm; mean ± SD)	2.0 ± 1.6	2.2 ± 1.7	0.93
CAL (mm; mean ± SD)	9.6 ± 2.6	6.3 ± 2.2	<0.01
Depth of intrabony defect (mm; mean ± SD)	5.1 ± 1.5	2.1 ± 1.0	<0.001

BoP, bleeding on probing; PPD, probing pocket depth; CAL, clinical attachment level; REC, gingival recession; SD, standard deviation.

**Table 3:** Correlation between SASP factors and clinical/radiographic outcomes at 12 months after surgery (Spearman Rho coefficient).

SASP Factor	PPD Reduction	*p* Value	CAL Gain	*p* Value	Bone Fill	*p* Value
IL-1β	−0.76	<0.001	−0.79	<0.001	−0.61	<0.001
IL-6	0.112	0.517	−0.008	0.963	0.11	0.540
IL-12	0.31	0.048	0.43	0.005	0.21	0.184
MMP-8	−0.68	<0.001	−0.69	<0.001	−0.61	<0.001
MMP-9	−0.64	<0.001	−0.72	<0.001	−0.57	<0.001

CAL, clinical attachment level; IL, interleukin; MMP, matrix metalloproteinase; PPD, probing pocket depth; SASP, senescence-associated secretory phenotype.

**Table 4:** Level of baseline clinical variables and SASP expression in sites achieving or not COM.

Variables	COM −	COM +	*p*-Value
PPD at baseline, mm (mean ± SD)	7.1 ± 2.1	8.3 ± 1.8	0.007
REC at baseline, mm (mean ± SD)	1.7 ± 1.0	1.6 ± 1.5	0.543
CAL at baseline, mm (mean ± SD)	8.8 ± 2.4	9.9 ± 2.5	0.125
Presence of plaque (%)	20.0	20.0	1
BoP (%)	66.7	44.0	0.165
N of predominant bony walls (1/2/3; %)	0.0/46.7/53.3	0.0/52.0/48.0	0.744
IL-1β (pg; mean ± SE)	1894.1 ± 195.7	849.8 ± 108.6	<0.001
IL-6 (pg; mean ± SE)	245.0 ± 146.8	47.2 ± 6.4	0.190
IL-12 (pg; mean ± SE)	181.7 ± 34.1	550.5 ± 129.3	0.015
MMP-8 (ng; mean ± SE)	455.8 ± 5.8	166.4 ± 12.3	0.001
MMP-9 (ng; mean ± SE)	134.2 ± 9.4	101.5 ± 37.1	<0.001

BoP, bleeding on probing; IL, interleukin; MMP, matrix metalloproteases; PPD, probing pocket depth; CAL, clinical attachment level; COM, composite outcome measure for the regenerative treatment; REC, gingival recession; SD, standard deviation; SE, standard error.

**Table 5:** Multiple logistic regression models show the relationships between significant baseline molecular mediators (classified as high and low according to the median level) and the composite outcome of periodontal regeneration (COM).

Variable	OR	95% CI	*p* Value
Model 1			
IL-1β (high vs. low)	0.02	0.00–0.50	0.016
MMP-8 (high vs. low)	0.06	0.01–0.65	0.021
Model 2			
IL-12 (high vs. low)	8.41	1.12–62.92	0.038
MMP-9 (high vs. low)	0.02	0.00–0.34	0.007

IL, interleukin; MMP, matrix metalloproteinase. Models were corrected for baseline probing pocket depth.

## Data Availability

The data that support the findings of this study are listed in the main manuscript.
